# Supplementary material for: Are carbon emissions trading and green financial instruments synergistic? -Comprehensive quantitative research based on content analysis
Source: PLoS One. 2024 Mar 7;19(3):e0298601. doi: 10.1371/journal.pone.0298601 (PMC10919601; doi:10.1371/journal.pone.0298601)
Supplement: S1 Data — (ZIP) [file pone.0298601.s001.zip › Data/地级市Z_F工作报告.zip!/╔╜╬≈/╠1⁄2╘¡╩╨/└┤╫╘╣1⁄2╓┌║┼í╛┬φ┐╦╩2╛▌═°í┐_2015.docx]

各位代表：

现在，我代表市人民政府向大会报告工作，请予审议，并请政协委员和其他列席人员提出意见。

一、2014年工作回顾

过去一年，面对国内经济下行压力加大的严峻形势，多重困难和挑战相互交织，我们在省委、省政府和市委的坚强领导下，在市人大、市政协的监督支持下，深入学习贯彻党的十八大、十八届三中、四中全会精神和习近平总书记系列重要讲话精神，以发挥“六个表率”作用为新的动力，努力工作，积极进取，在化解矛盾中稳定大局，在推进改革中寻找动力，在调整结构中保持增长，在优先投入中改善民生。

——经济增长和结构调整有新成效。实现地区生产总值2531.09亿元，增长3.3%；固定资产投资1746.09亿元，增长4.5%；社会消费品零售总额1411.13亿元，增长10.1%；一般公共预算收入258.85亿元，增长4.7%；城镇常住居民人均可支配收入25768元，增长7.9%；农村常住居民人均可支配收入12616元，增长10.4%；两个收入增长均快于经济增长。省市重点工程完成投资1360.69亿元，为年度目标任务的108.5%。经济结构有新的优化，新兴接替产业增加值、服务业增加值、非煤产业增加值占比分别提高到59.8%、58.5%、85.1%。江铃重汽、阳煤化机、北车太原铁路装备园等重大项目进展顺利，有的已投产见效。富士康太原园区投资32亿元，新上苹果iphone5s智能手机装配线，完成工业总产值482.56亿元，增长21.4%。高新技术企业数量增长35.8%。民营经济保持良好发展势头，对经济增长和社会就业的贡献更加突出。

——改革开放有新进展。深化行政体制改革，探索“六权治本”模式，推行权力清单制度，加强对权力运行的制约和监督。简政放权，取消、调整、下放行政审批事项119项，审批时间压缩21.6%；全年受理审批事项近10万件，办结率99.6%。实行全口径预算管理和预决算公开，市级117个部门全部公开部门预算和“三公经费”预算。深化工商登记制度和农村土地制度等各项改革，激发各类市场主体发展活力。完善扶持中小微企业发展政策，深化政银企助保贷制度，为企业发展“施肥增养”。开发区基础建设和开放招商引资有新进展，清华大学创业园、润恒农产品冷链物流等重点项目进展顺利。

——城市基础设施建设有新突破。推进城市快速交通体系建设。继中环建成后，实施建设路、长风街、南沙河路快速化改造建设，快速路网体系日趋完善。完成全长21公里阳兴大道快速路建设，缩短了市县连接时空距离。改造长治路、太榆路等25条道路，改造小街小巷27条，总长139公里。铺设供水、排水、雨水、电力、通讯、供热、燃气地下管线745公里。城南20万吨污水处理厂建成投运。更新清洁能源公交车600辆，新开通公交线路5条。公共自行车达到4.1万辆。太原南站候车楼及地下停车场、西广场、主要路网全面竣工，并于7月1日正式投入使用。以城郊森林公园建设为重点，加大东西两山生态建设力度，完成营造林27.91万亩。建成区绿化覆盖率达到40.5%，绿地率达到35.57%，人均公园绿地达到11.26平方米。

——省城环境质量有新提升。推进“五大工程、五项整治”，省城环境质量持续改善。集中供热扩网2500余万平方米，拆除燃煤锅炉465台。拆除城中村279万平方米，拔掉黑烟囱6000根。拆除棚户区113万平方米，取缔小火炉12465台。启动40个城边村气化改造，24个村实现通气。大力推进常年运行燃煤锅炉关停或改造。关停东山电厂等污染企业30个。强制报废黄标车和老旧机动车38229辆。6项主要污染物减排指标超额完成省定任务。集中式饮用水源地水质达标率稳定保持100%。广泛开展城乡清洁工程，省城主要街道全部实现机扫。全年市区优良天数197天，较上年增加35天；空气质量综合指数下降11.5%。

——民生改善有新变化。着力提升托底保障能力，民生支出270.66亿元，占全市一般公共预算支出的83.9%，比上年提高1个百分点。城镇新增就业10.55万人，城镇登记失业率控制在3.4%。36.8万企业退休人员月平均增资238元，全市最低工资标准平均增幅达13%以上。完善社会救助体系建设，加强社区惠民服务。推进教育医疗资源标准化、均衡化、优质化建设，太原幼儿师范学校和市人民医院等开工建设。新建开工保障性住房48270套、基本建成25375套，改造农村危房6500户。大力发展文化事业，县级文化馆、图书馆和乡镇（街道）文化站实现全覆盖。成功承办省第十四届运动会，取得全省第一的优异成绩。严格落实安全生产责任，全市未发生重特大安全生产事故。圆满完成第三次经济普查工作。加强社会综合治理，保持和谐稳定社会局面。

各位代表，一年来取得的成绩，是省委、省政府和市委正确领导的结果，是市人大、市政协监督支持和全市人民共同努力的结果。在此，我代表市人民政府，向全市人民，向中央、省属驻并单位和企业，向各民主党派、工商联、无党派人士、人民团体，向人大代表和政协委员，向驻并部队和武警官兵，向所有关心支持太原改革发展的社会各界人士，表示崇高的敬意和衷心的感谢！

回顾过去的一年，我们也清醒地看到前进中的困难和挑战。投资增长乏力，经济仍在低速运行；园区引领能力不强，好项目、大项目不多的老问题没有破解；社会管理创新不够，维护稳定的压力加大；民生改善欠账较多，城中村改造存在许多困难和问题；政府工作存在不足，职能转变还需加强；“四风”问题尚未根绝，不作为乱作为、懒政怠政、失职渎职依然存在；我省发生系统性、塌方式腐败，面临政治、经济、民生、生态环境立体性困扰；少数政府机关工作人员腐败问题严重，党风廉政建设和反腐败斗争任务艰巨。我们将积极面对、勇于担当，认真解决存在的问题。

二、2015年工作安排

今年是全面深化改革、全面推进依法治国的关键之年，是全面完成“十二五”规划的收官之年，也是我市以“五个一批”为载体、发挥“六个表率”作用的推进之年。政府工作的总体要求是：深入贯彻党的十八大、十八届三中、四中全会精神和省委、市委十届六次全会精神，贯彻落实习近平总书记系列重要讲话精神，按照“四个全面”战略布局，主动适应经济发展新常态，坚持稳中求进工作总基调，以提高经济发展质量和效益为中心，把转方式调结构放到更加重要位置，全面深化改革，扩大开放，突出创新驱动，强化风险防范，保障改善民生，围绕“六个表率”要求，着力抓好“五个一批”，全力推进“六大发展”，认真做好“十三五”规划编制工作，谋划好未来发展，促进全市经济平稳健康发展和社会和谐稳定。

经济社会发展的主要预期目标是：地区生产总值增长7%左右，固定资产投资增长16%，社会消费品零售总额增长10%，一般公共预算收入增长7%，城镇常住居民人均可支配收入增长7%，农村常住居民人均可支配收入增长8%，居民消费价格涨幅控制在3%左右，城镇新增就业人数10.3万人，城镇登记失业率控制在4%以内。

约束性指标是：万元地区生产总值综合能耗下降 2.8%，单位工业增加值用水量和二氧化硫、化学需氧量、氮氧化物、氨氮、烟尘和工业粉尘排放量完成省下达任务，PM2.5年均浓度值下降4%。

（一）主动适应和引领新常态，推进产业结构调整和转型升级

坚持创新引领发展。创新是引领发展的第一动力。适应和引领经济发展新常态，关键是要依靠科技创新转换发展动力。抓创新就是抓发展，谋创新就是谋未来。要强化战略提升、管理变革、产品升级、产业转型的核心理念，积极实践和丰富“互联网+”理念和行动计划，推动移动互联网、云计算、大数据、物联网等与现代制造业结合，促进电子商务、工业互联网和互联网金融健康发展，寻找更多的发展机遇和更大的发展空间。企业是创新的主体。要加大企业科技创新投入，强化创新驱动，驾驭新常态。推进实施富士康太原园、江铃福特重汽配套园区千亿元计划，抢占新兴产业和高端制造业竞争高地，形成现代信息产业和现代制造业集聚效应，带动太原产业转型升级。

推进信息化和工业化深度融合。探索和创新工业4.0“中国版”新思维和新路径，培育和发扬极致“工匠”精神，着力打造矿用成套设备、重型机械、铁路和化工、电力装备、新能源汽车、不锈钢深加工等产业集群，重点抓好阳煤现代煤化工装备制造基地、中天信安防科技、太原锅炉厂、太重轮对、北车工程机械等一批领军项目。实施“中国制造2025”，坚持创新驱动、智能转型、强化基础、绿色发展，建设制造强市，打造太原制造品牌，实现低成本、高质量、新生态、可持续发展。加大现代信息技术对传统产业的改造，强化互联网思维，推动煤电等传统产业提质升级。认真贯彻省政府为企业减负60条新举措，促进全市工业经济平稳运行。

积极推进开发区扩容提质。把高新区、经济区、民营区、不锈钢园区作为领先发展的重要载体，切实发挥好综合保税区对外开放的重要作用。实施北扩南延战略规划，近期拓展60平方公里左右，为开发区快速发展创造空间载体。深化开发区体制机制改革创新，靠改革增动力、开放拓空间。强化对开发区经济增长贡献、投资开发强度、招商引资质量数量等主要业绩考核，树立发展导向。做大做强县域经济，创造区域特色优势。

大力发展现代服务业。发展现代服务业是社会经济转型升级的内在要求，也是省会城市的资源和区位优势。主动顺应消费需求升级，重点发展智力化、资本化、专业化、效率化的现代服务业，重点发展和培育总部经济、物流快递、电子商务、现代金融等产业，逐步增强服务业的支撑作用。抓好华润万象城、万达城市综合体、山西国际金融中心、汾酒集团总部经济、中海和绿地集团楼宇经济等重大服务业项目，向以服务业为主体的第三产业要动力。积极培育消费热点，支持社会力量兴办各类服务机构，发展养老、健康、文化等服务业，满足人民群众多样化的服务需求。

培育壮大都市现代农业。加快转变农业发展方式，科技创新，产品升级。适应城郊型特点，建设高质量“菜篮子”、“米袋子”、“奶瓶子”基地。利用资源禀赋，发展生态休闲观光、健康养生等特色农业。抓好宝迪养殖屠宰加工一体化、九牛现代农业循环产业园、康培农业科技示范园等龙头项目，打造精品化、现代化、高效化新型农业。扎实推进农村土地承包经营权确权登记颁证工作。积极开展美丽示范乡村创建活动。继续推进百企千村产业扶贫开发工程，组织单位定点扶贫、党员干部结对帮扶，实施精准扶贫，力争脱贫1.3万人。

（二）全面深化改革开放，不断增强发展的活力和动力

改革开放是推动发展的制胜法宝。要最大范围凝聚改革力量，最大能量释放制度红利，最大限度激发创造活力。要按照习近平总书记“人民有所呼，改革有所应”的要求，坚持“改革为了人民、改革依靠人民、改革成果让人民共享”的理念，从人民利益出发谋划改革、推进改革，让人民从改革中得到看得见、摸得着的实惠，增强人民群众的获得感。

加大简政放权、放管结合改革力度。按照中央和省的统一部署，再取消和下放一批审批事项，全部取消非行政许可审批，建立规范行政审批的管理制度。建立市场准入负面清单，公布市级政府权力清单、责任清单，切实做到法无授权不可为、法定职责必须为。市、县政府对应当放给市场和社会的权力，要彻底放、不截留，对中央和省下放的审批事项，要接得住、管得好，真正做到企业的事情交给企业、市场的事情交给市场、社会的事情交给社会，创造公平竞争、开放创新的环境。

加快行政审批制度改革，建设综合性政务服务平台。真正落实“一口受理”、实行限时办理、严格规范办理、坚持透明办理、推进网上办理，为企业高效服务，为创业提供便利。大幅减少投资项目前置审批，大幅缩减政府核准投资项目范围。在城市规划控制区范围内，教育、卫生、养老、办公等公共服务类项目和商品住宅、写字楼、购物中心等商业开发项目审批，取消土地预审、矿产覆压、水土流失等前置性条件，大幅简化审批程序，用政府权力的“减法”、依法办事的“加法”，换取市场活力的“乘法”。

全方位推进开放。开放是强市之路，以开放培育和催生经济社会发展新动力，赢得转型发展的主动、市场竞争的主动。以开放释放创造力、生产力，推动大众创业、万众创新，让千千万万个市场细胞活跃起来，汇聚成发展的巨大动能。下大力气招商引资，引进聚集一批行业领先、带动性强的大企业、好项目，引进一批先进技术和高端人才，富集加快发展的资源要素。民营经济是开放的产物，蕴藏着无穷的创造力，要倾力解决其发展中的体制性障碍、制约性瓶颈、政策性问题，充分释放企业家的创新精神和能力。小微企业是最大的生力军、有生力量，要留足市场发展空间，加快中小微企业园区建设，搭建公平竞争舞台，厚植创业创新文化，更好地实现创业发展和自身价值。要适应互联网时代的“网状组织”，积极推进“企业平台化、员工创客化、用户个性化”，以组织构架和市场模式的变革，迎接互联网时代的挑战。

（三）加强城市基础设施建设，改善省城环境质量

城市是我们的精神家园和发展载体。让出行更方便、环境更宜居、城市更美好、人民更幸福，是永恒的追求和目标。

加大城市基础设施建设力度。道路是城市的血脉，是城市流动的风景线。道路畅通是现代城市的基本条件。改造南内环、学府街、兴华西街，续建南沙河快速路、龙城大街东延等主次干道，尽快启动滨河东路南延工程，改造小街小巷30条，完善中环路、建设路、长风街、滨河东西路等快速路重要节点，构建快速高效、级次匹配、路网完整的道路交通体系。加快南客站、太原站东广场建设，强化功能配套，完善周边路网，提升服务水平。优化线型和空间布局，推进地铁2号线建设。加快建设500千伏龙城供电及送出等电力工程，完善电网输变体系。建设晋阳32万吨污水处理厂、4500吨垃圾焚烧电厂、餐厨垃圾、污泥无害化处理等重点项目，完善污水处理和垃圾收运处理体系。

加强城市综合整治。坚持建管并重，不断提升城市管理水平和城乡规划建设水平。建立健全城市管理体制，按照属地管理、依法行政和责权利统一原则，权力下放，重心下移。大力度加快城市静态交通建设。创建国家“公交都市”，推广应用新能源公交车辆。加强县城和中心镇建设，加大市政公用和公共服务设施建设力度，提升品质，改善环境。加强城乡统筹，搞好村居环境特别是城边村的综合整治，改善农村人居环境。健全完善城市信息资源，开展地下管线综合普查，建立高效集成的信息共享平台，提高城市管理数字化、规范化、精细化、常态化水平，建设智慧城市。

强化省城环境质量改善。良好的生态环境是最公平的公共产品，是最普惠的民生福祉。认真落实控制燃煤、关停污染企业等改善环境的十大措施，深化“五大工程、五项整治”，二级以上优良天气力争达到60%以上，实现环境质量全面好转的阶段性目标。加快大气污染治理步伐，以集中供热和清洁能源转换为手段，多措并举整治燃煤污染，加快古交兴能电厂一二期超低排放改造、三期2×66万千瓦热源电厂建设、太古热源长输管网建设，确保2016年实现供热5000万平方米。加快推进交城晋能热源长输管网建设和华能东山燃气热电及配套管网建设，力争实现新增集中供热面积3000万平方米，替代既有建筑2100万平方米。实施30个城边村气化改造和未改造城中村洁净煤置换，大幅度减少冬季燃煤总量。关停搬迁主城区污染企业，完成太钢电厂和二电厂超低排放改造，加快一电厂迁建工程、瑞光热源电厂建设工程，全方位推进重点企业环境综合整治。严格控制面源污染，严格执行施工现场绿色标准，坚决控制扬尘污染，禁绝秸秆垃圾焚烧，从严整治露天烧烤，依法淘汰黄标车和老旧机动车1.15万辆。全面启动娄烦水源地保护工程。加强水污染治理，集中式饮用水源地水质达标率稳定保持100%。

加大生态文明建设力度。完成营造林29.07万亩。建设东西山绿色生态屏障。新建北山森林防火通道59.4公里，形成东、西、北山完整的森林防火通道体系。开工建设晋阳湖、太山植物园、和平公园等城市公园和汾河南部景观三期工程，凸现太原“三面环山、一水中分”的山水城市大格局。

（四）切实保障和改善民生，让发展成果更多惠及人民群众

以增进民生福祉为追求，实施一批重大民生项目，全面加快城乡社会事业发展，促进社会公平正义与和谐进步。

全力推动城中村改造。加快城中村改造，既是重大民生工程，又是重大发展工程。要加大整村拆除力度，同步实施安置房建设，落实拆迁谁、改变谁、造福谁的惠民目标。要以规划引领为先导、区级政府为主体、惠民政策为根本、平安拆迁为底线、合作团队为关键、审批效率为保障，牢牢把控社会风险、资金风险和市场风险。同步规划教育、文化、体育、医疗、社会管理、社区服务中心等公共服务设施，同步配套水电气暖、道路、绿地、停车场、环卫等基础设施，高度重视历史文化遗存保护，大力发展、培育和保护特色产业，举全市之力，坚决打赢城中村改造这场硬仗，变城市的暗点为城市的亮点，实现省会城市的华丽转身。

坚持把就业作为民生之本。要加强就业指导和创业扶持，认真落实促进就业的各项政策，鼓励创业带动就业。做好高校毕业生等重点群体的就业工作，加大对残疾人、零就业家庭等困难人员的就业援助力度。全面治理拖欠农民工工资问题，健全劳动监察和争议处理机制，让法律成为劳动者的守护神。

推进教育医疗资源标准化、均衡化、优质化发展。深化教育领域综合改革，调整和优化中小学、幼儿园布局，大力推进现代职业教育改革和发展，在优化调整专业设置基础上建设职教园区，鼓励企业和社会发展职业教育。续建52中、64中、财校等9所学校，新建太原五中、成成中学、十二中等11所学校，积极发展公办、民办等多元体制幼儿园，努力满足社会需求。加快推进山西大学东山新校区建设。完善太原大学配套功能，不断提升教育质量和影响力。加大公立医院改革力度，促进优质医疗资源向基层流动，规范配建社区卫生服务机构，为群众提供质优价廉的医疗卫生服务。搭建市级健康云平台，推进市中心医院、市妇幼保健院、市人民医院和市公共卫生中心新建工程。加强公共体育设施建设，继续做好人口和计划生育工作。

进一步健全完善社会保障体系。不断扩大社会保险覆盖面，加快城乡社会保险一体化发展，坚守民生保障底线。大力度推进采煤沉陷区综合治理。办好农村饮水安全和危房改造等民生实事。加快棚户区改造和保障性住房建设，新建开工保障性住房57572套，基本建成42300套。用足用活住房公积金，为改善住房条件和稳定房地产市场加力。推进既有建筑节能改造，让人民群众增加温暖过冬的幸福指数。开工建设老年社会福利院、社会（儿童）福利院等民生项目，发展社区和居家养老事业，推动社区便民服务提档升级。城乡居民最低生活保障标准在现有基础上分别提高5%、6%。继续实施“一元菜”惠民政策。推进农村公路完善提质工程。

牢固树立安全发展理念。把安全生产作为最大民生、比泰山还重的责任、任何人任何时候任何地方都不可逾越的红线。健全“党政同责、一岗双责、齐抓共管”的责任体系，落实政府安全监管责任，强化企业安全生产主体责任，严格落实安全生产“一票否决”制，加强对煤矿、燃气、公共场所、人员密集场所等重点部门和环节的安全检查和监管，切实消除事故隐患，坚决杜绝重特大事故，减少一般性事故。加大食品药品安全监管力度，确保人民群众舌尖上的安全。

加强和创新社会治理。全力维护社会稳定，推动政府治理和社会自我调节、居民自治良性互动。深入开展矛盾纠纷排查化解工作，及时妥善处置突发性群体事件。继续保持打黑除恶的高压态势，全力推进以“天网”视频监控为主的社会治安防控体系建设，努力营造和谐稳定的社会环境。健全普法教育机制，继续深入开展“法律六进”活动。支持工会、共青团、妇联等人民团体发挥作用。建立未成年人社会保护制度，为农村留守儿童、妇女、老人提供关爱服务。加强应急队伍、应急能力建设。加强气象防灾减灾能力建设。加强国防后备力量建设和双拥工作。

（五）促进文化事业发展，提升历史文化名城影响力

文化是城市的灵魂，城市是文化的载体。要传承优秀的历史文化，创造高雅的现代文化，让人民群众享有更多文化发展成果。

加大文化旅游产业发展力度。加快历史文化街区保护和修复，保护好府城普光寺、文殊寺、关帝庙等历史遗存，综合整治天主教堂、纯阳宫、崇善寺、清真古寺等国保单位的周边环境，延续历史文脉，守护文化尊严，留住城市记忆。积极推动钟楼街步行街保护性改造，融入现代元素，打造传统与时尚交融、老字号与新业态混合、历史传承与商业价值并重的城市新名片，增强市场活力和文化魅力。不断提升“唐风晋韵·清凉太原”的知名度和影响力，推进晋祠大景区、太山龙泉寺景区、阳曲青龙古镇、晋源农耕文化等保护工程，发展太化工业遗址创意文化产业园，形成历史文化名城的产业支撑，培育新兴文化产业的增长点。

巩固文化体制改革成果。培育和践行社会主义核心价值观，以创建全国文明城市为引领，推动“五城联创”，全面提升城市两个文明建设水平。将太原古老厚重的历史文化与城市核心价值观紧密结合，以良好的公共文化基础设施、丰富的文化活动，保障人民群众基本文化权益，满足人民群众不断增长的文化需求。打造一批精品力作，讲好太原故事，提升文化传播的影响力。加快市图书馆改扩建工程，力争年底竣工使用。以承办全国第二十五届图书交易博览会为契机，组织“书香太原”、“电影下乡”和“锣鼓大赛”等系列文化活动，开创文化发展的新局面。

（六）切实加强政府自身建设，提升为民服务能力

做好今年的各项工作，必须进一步加强政府自身建设，全面推进依法行政，讲政治、讲规矩、守纪律，建设法治政府、廉洁政府、服务型政府，增强政府执行力和公信力。

深化“六权治本”，把权力关进制度的笼子里。坚持依宪施政、依法行政，把政府工作全面纳入法治轨道，让法治筑牢社会共识的基座，以制度管权、管钱、管事。要尊法学法守法用法，依法全面履行职责，所有行政行为都要于法有据，任何行政部门都不得法外设权。要强化法治思维，努力形成办事依法、遇事找法、解决问题用法、化解矛盾靠法的良好法治环境。要自觉接受人大及其常委会的监督，接受人民政协的民主监督，加强行政监察和审计监督，认真听取人大代表、政协委员、各民主党派、工商联、无党派人士和人民团体的意见，办理好人大代表建议和政协委员提案，全面接受人民的监督，充分体现人民的意愿。

强化为民宗旨，坚持“三严三实”。巩固学习讨论落实活动和党的群众路线教育实践活动成果，继续完善并严格执行作风建设各项制度，积极开展“三严三实”专题教育，把讲认真、抓落实作为政府工作常态，把强化责任、靠前指挥作为政府工作方法，努力在深化“四风”整治上见实效，在守纪律讲规矩、营造良好政治生态上见实效，在真抓实干、推动改革发展稳定上见实效。大力弘扬敢于担当、积极作为、迎难而上的精神，建设为民务实清廉的公务员队伍。

深入推进党风廉政建设和反腐败斗争。牢固树立“抓党风廉政建设是本职、不抓是渎职、抓不好是失职”的理念，全面贯彻政府系统党风廉政建设责任制和党委党组主体责任，自觉履行领导班子成员“一岗双责”。认真执行中央“八项规定”，持续纠正“四风”突出问题，严厉惩处顶风违纪现象，坚决整治损害群众利益的不正之风。始终保持惩治腐败的高压态势，对腐败分子零容忍、严查处，形成不敢腐、不能腐、不想腐的机制。要加强理论学习和党性修养，是非明于学习、境界升于自省、名节源于修养、腐败止于正气，筑牢拒腐防变的思想堤坝。

各位代表，做好2015年工作，任务艰巨，责任重大。让我们紧密团结在以习近平同志为总书记的党中央周围，在省委、省政府和市委的领导下，以高度的党性自觉、历史的责任担当、顽强的拼搏精神，扎实做好“五个一批”落实工作，为实现“六个表率”、推动“六大发展”、促进富民强市而努力奋斗！
